# The mitochondrial genome of *Phyllodiaptomus tunguidus* Shen & Tai, 1964 (Copepoda, Calanoida) and its phylogeny

**DOI:** 10.1080/23802359.2020.1763863

**Published:** 2020-06-22

**Authors:** Xiaoli Zhang, Shaolin Xu, Ping Liu, Qi Huang, Huiming Li, Henri J. Dumont, Bo-Ping Han

**Affiliations:** a Department of Ecology, Jinan University, Guangzhou, China; b Department of Biology, University of Gent, Gent, Belgium

**Keywords:** Freshwater copepod, *Phyllodiaptomus tunguidus*, mitogenome, phylogenetic analysis

## Abstract

The mitochondrial genome of *Phyllodiaptomus diaptomus* was sequenced and assembled via Next-Generation Sequencing (NGS) and iterative assembly with a reference seed. The genome is 16446 bp long, A + T biased (69.4%), with 13 protein-coding genes, 22 tRNAs, and 2 rRNA. All protein-coding genes are initiated by a typical “ATN” codon. ND1, ND2, ATP6 genes are terminated with “TAG”, the other 10 genes are terminated with “TAA”. This is the first complete mitogenome published in the Diaptomidae. It provides molecular information useful to a better understanding of the phylogeny of calanoids.

*Phyllodiaptomus tunguidus* is a Chinese endemic and was first described by Shen and Tai (1964) from the specimens collected from the Pearl River Delta in southern China. The original specimens of the species were stored in Zoological Museum, Institute of Zoology, Chinese Academy of Sciences (CAS). It belongs to the Calanoida, Diaptomidae and is mainly distributed in South China. *P. tunguidus* is a dominant species of zooplankton in most reservoirs of tropical and subtropical China (Lin et al 2003; Zhao and Han 2007). So far only one species of this genus has been found in China. Due to its large size and abundance, it has a strong effect on phytoplankton and it therefore plays an important role in tropical freshwater food webs. Complete mitochondrial genome data of such keystone species provide useful information for understanding its own phylogeny and the ecology of the order Calanoida (Jooste et al. 2019). Here, we sequence and annotate the mitochondrial genome of *P. tunguidus* and compare it to that of other copepods.

Living animals were collected from Liuxihe reservoir (113.46°E, 23.76°N) and fixed with absolute ethanol. The specimens (accession number COZOOP03003A) and extracted DNA were preserved at −20 °C in the Aquatic Collection of Institute of Hydrobiology, Jinan University, Guangzhou, China. We used the TIANamp Marine Animals DNA Kit (TIANGEN BIOTECH CO., LTD) to extract the genome. Genomic DNA was sequenced using a next-generation method. The assembly and annotation procedure followed Xu et al. (2018) with the COI sequence of *P. tunguidus* as seed. The mitochondrial genome was annotated with the MITOS WebServer (Bernt et al. 2013) and verify via BLAST (Altschul et al. 1990). Transfer RNA genes were double-checked with tRNAscan-SE v.2 (Lowe and Eddy 1997) and ARWEN V.1.2.3 (Laslett and Canback 2008). A phylogenetic tree was constructed on 13 PCGs of 6 available mitogenomes of three Copepoda taxa from NCBI. Due to extensive gene reshuffling in this group (Minxiao et al. 2011) the sequences were separated into protein-coding genes, which were aligned separately via AliView (Larsson 2014). A Bayesian phylogenetic tree was constructed using a JTT substitution model in BEAST2 (Bouckaert et al. 2014). Finally, the synteny of all mitochondrial genes between *P. tunguidus* and *Lovenula raynerae* was analyzed using SimpleSynteny (Veltri et al. 2016).

The total length of the *P. tunguidus* mitogenome is 16446 bp (GenBank accession number: MN927223, released on March 8, 2020), with A + T biased composition: A (34.6%), C (12.3%), G (18.2%), T (34.8%). Thirty-seven genes were annotated: 13 protein coding genes, 22 tRNA genes and 2 rRNA genes. All protein-coding genes are initiated by a typical “ATN” codon. COX1, COX2, COX3, ND2, ND3, ND4, ND5, ND6, ND4L, CYTB are end with TAA; ND2, ND1 and ATP6 end with TAG; tRNA genes have lengths ranging from 56 to 68bp, and they are folded into a typical cloverleaf structure. The Phylogenetic tree shows that copepods are split into three orders-level clades, namely Calanoida, Cyclopoida and Harpacticoida (Figure 1). *Phyllodiaptomus tunguidus* is monophyletic with *L. raynerae* and the two calanoids have a sister taxon relationship with *Calanus hyperboreus*. Compared with the mitogenome of *L. raynerae*, a different arrangement of tRNAs was found, but no shift of PCGs was discovered.

**Figure 1.**
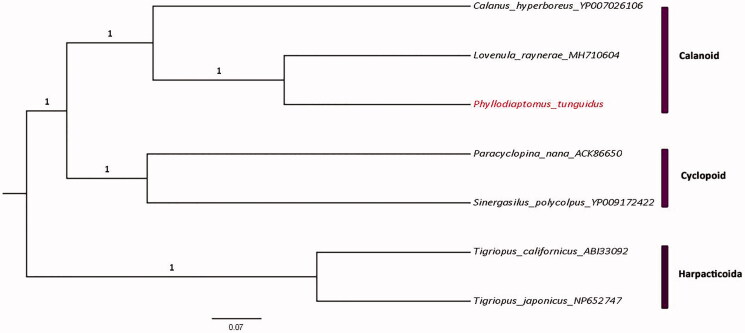
Bayesian phylogenetic tree of *Phyllodiaptomus tunguidus* and 6 other copepods species. The numbers next to the nodes are posterior probabilities. The phylogeny was reconstructed based on 13 mitochondrial PCGs via Beast.2 with ngen set to 10 million.

## Data Availability

The data that support the findings of this study are openly available in NCBI at https://www.ncbi.nlm.nih.gov/nuccore/MN927223.1, GenBank number: MN927223.1.
